# Co-Creating Recommendations to Redesign and Promote Strength and Balance Service Provision

**DOI:** 10.3390/ijerph16173169

**Published:** 2019-08-30

**Authors:** Calum F Leask, Nick Colledge, Robert M E Laventure, Deborah A McCann, Dawn A Skelton

**Affiliations:** 1 Aberdeen City Health & Social Care Partnership, Marischal College, Broad Street, Aberdeen AB10 1AB, UK; 2 Health Intelligence Department, NHS Grampian, Summerfield House, Eday Road, Aberdeen AB15 6RE, UK; 3 External Consultant, Robin Park Sports Centre, Loire Drive, Newtown, Wigan WN5 0UL, UK; 4 Later Life Training, Silver Cottage, Main Street, Killin FK21 8UT, UK; 5 Inspiring Healthy Lifestyles, Robin Parks Sports Centre, Loire Drive, Newtown, Wigan WN5 0UL, UK; 6 Physical Activity Exchange, School of Sport and Exercise Sciences, Liverpool John Moores University, 70 Great Crosshall Street, Merseyside L3 2AB, UK; 7 School of Health and Life Sciences, Glasgow Caledonian University, Cowcaddens Road, Glasgow G4 0BA, UK

**Keywords:** Co-creation, physical activity, leisure services, service redesign, participatory, older adults, strength, balance

## Abstract

*Background:* Awareness of physical activity guidelines are low, particularly the “forgotten guidelines” of strength and balance. Increasing awareness of guidelines, but also of appropriate local services that can be utilised, is an important step towards active ageing. Co-creation can inform tailored service provision to potentially increase uptake and adherence. The aim was to co-create recommendations to redesign and promote local leisure services, emphasising strength and balance activity provision. *Method:* Twenty-four ageing and older adults engaged in 10 co-creation workshops. Workshops consisted of interactive tasks, and fieldwork tasks were undertaken externally. Data were collected using field notes, worksheet tasks and facilitator reflections and were analysed using qualitative content analysis. *Results:* Retention and adherence rates were 92% and 85%. Co-creators cited group cohesion, scientific input from experts and perceived knowledge development as enjoyable elements of the process. Four key themes emerged from analysis: (1) localised strategies for awareness raising, (2) recruitment of volunteer champions to increase uptake and maintenance, (3) accessibility of activities, including what they are and when they are, and (4) evaluation of impact. *Conclusion:* This has been the first study, to our knowledge, to utilise co-creation for informed leisure service provision improvement. Future work should aim to implement these recommendations to ascertain what impact these themes might make.

## 1. Introduction

Despite the significant contribution that physical activity (PA) has on maintaining health and wellbeing in later years, levels of PA rapidly decline with age [1]. Despite the importance of all domains of PA for adults and older adults, within PA, guidelines for health, muscle and bone strengthening and balance activities (MBSBA) are considered ‘the forgotten guidelines’ [2,3]. Fewer than half of individuals who reach the cardiovascular recommendations meet the strength and balance guidelines, thus demonstrating a need to promote the importance of MBSBA throughout the lifecourse [4,5,6]. Recent evidence has suggested that progressive resistance training has favourable effects not only on muscular strength, physical capacity and the risk of falls, but also that its benefits extend to cardiovascular function, metabolism and coronary risk factors [6,7]. 

Notwithstanding the PA guidelines and this growing evidence base, there has been limited dissemination of this literature and its implications for wellbeing through public engagement. Increasing the population’s awareness and knowledge of these guidelines is an important first step towards improved outcomes [8]. However, physical activity literacy can be poor, particularly in understanding the differences between components of fitness, such as aerobic activity and strength activities and their effects on health outcomes [9]. Innovations such as the Functional Fitness MOT (FFMOT) aim to increase older peoples’ knowledge and understanding of the different components of fitness and the different local activities and programmes that can improve these. A greater appreciation of physical literacy can lead to changed health behaviours; for example, 25% of people sought out new exercise opportunities after completing their FFMOTs [10]. 

To develop strategies to ensure that information is adequately disseminated, in addition to ensuring that services are designed to the specific needs of users, it is increasingly advocated to engage the target population in these initiatives [11]. The process of co-creation, which may be defined as collaboration between different groups of stakeholders to develop a mutually beneficial outcome, has been shown to increase individual satisfaction, improve awareness of individual health behaviours, improve the effectiveness of programmes and enhance the quality of service provision [12,13]. Emerging evidence has demonstrated that co-creation can be used with older cohorts to create public health solutions [13], however, it has had limited utilisation in the development of awareness raising strategies and to inform provision of PA opportunities.

The aim of this study was to utilise co-creation with ageing and older adults to develop recommendations for redesigning and promoting local leisure services with an emphasis on MBSBA. 

## 2. Materials and Methods

### 2.1. Study Design

A series of co-creation workshops was undertaken in both a leisure and community setting to explore recommendations and improvements that could be made to leisure services locally. A variety of stakeholders engaged in this process, including: end-users, researchers specialising in co-creation and strength and balance, and public health officers. The process was facilitated by four individuals, who received training from experts in co-creation theory to ensure effective delivery of, and engagement within, the workshops. Two groups of end-users were recruited, one aged 65 and over and one aged 50–65 years. Both groups completed 10 workshops between April and July 2018, with each workshop lasting a maximum of 150 minutes. Fieldwork tasks were also administered and completed between workshops. The process was iterative, whereby important points of discussion could be revisited based on the needs and preferences of the co-creators. As data collection fell within the remit of service redesign, ethical approval was not required. 

### 2.2. Participants

There were 24 end-users recruited onto the programme—12 aged 50–65 years for the pre-retirement group (hereafter referred to as the Wigan group) and 12 aged over 65 for the post-retirement group (hereafter referred to as the Leigh group). Collectively, these individuals were branded as the ‘New Tricks Research Group’ to support the development of ownership and facilitate a sense of belonging and purpose at an early stage [14]. Both groups were collectively recruited through a combination of workplace advertisements, community networks and social media. Inclusion criteria for individuals were: aged within the pre-defined parameters, community-dwelling, able to give informed consent and able to attend a minimum of nine workshops. Once individuals expressed an interest in engaging in the project, information packs were distributed. Incentives were also provided to encourage engagement, including a free physical activity tracker and a free annual leisure membership. A purposeful sampling approach was adopted [15] to ensure that the participants engaged were representative of the end-user population. Specifically, the demographic information collected on which this was judged included: activity status, gender, age, medical conditions, disability and socio-demographic profile (derived from postcode data). All individuals volunteered to engage and ethical standards were adhered to, including: data were treated confidentially at each stage, no individuals were identifiable in the results and all participants were reminded that they could withdraw at any time without consequence.

### 2.3. Co-Creation Workshops

The contents of each workshop are summarised in Table 1. The original plan for this project was semi-structured to reflect the recognition that the needs and preferences of the co-creators may change over time and therefore, the structure of the programme may have to be agile to adapt to these assumptions. 

Key principles in the development, implementation and evaluation of co-creation projects [16] were followed during this process. Important considerations included:

Sampling—in addition to purposefully sampling co-creators across the spectrum of demographic criteria, interactive fieldwork tasks were also used as an informal form of snowball sampling to gain a wider variety of opinions and perspectives to inform the process [17].

Ownership—further to branding the group, strategies were incorporated throughout to facilitate ownership of the process. These included inviting co-creators to present their findings at the stakeholder event in week 10, in addition to providing opportunities to contribute to other local programmes to promote healthy ageing [14].

Defining the procedure—central to the process was the up-skilling of all co-creators to ensure an even platform of knowledge as a basis for informed decision making. For example, whilst academic co-creators up-skilled others on the benefits and measurement techniques around strength and balance, end-user co-creators up-skilled others regarding their needs and preferences. Further techniques derived from action research that were incorporated here included brainstorming (compiling a spontaneous list of ideas) and ranking tasks (in order to prioritise ideas) [16,18].

### 2.4. Data Collection

Data were collected using worksheet tasks, in-depth field notes taken during workshops for continuous member-checking [19], summary reports at the end of sessions and reflective logs from facilitators to capture key learning and implementation considerations for future workshops. To determine satisfaction of engagement, workshop attendance, retention rates, and satisfaction questionnaires were distributed. Self-reported physical activity was collected at baseline using a single item questionnaire.

### 2.5. Data Analysis

As the purpose of this research was to categorise different solutions that could be utilised to improve leisure services, qualitative content analysis was completed manually, similar to previous co-creation projects [13]. Summary reports, detailed field notes during discussions, workshop tasks and reflective logs written by facilitators were the units of data that were analysed, following a multi-phased approach [20]: (1) Determine the research question, (2) Select appropriate materials, (3) Develop a coding frame and split data into coding units, (4) Conduct analysis, (5) Interpret and present the findings. 

The process was evaluated by continuous member checking on all discussion topics and generated summary reports to ensure that documented findings were representative of co-creators’ opinions. These findings were cross-referenced by all members of the authorship team before again being member checked with all co-creators for accuracy in a final report form.

## 3. Results

### 3.1. Participant Profile 

The profile of the individuals recruited into the workshops is reported in Table 2.

### 3.2. Attendance and Retention

**Figure 1:**
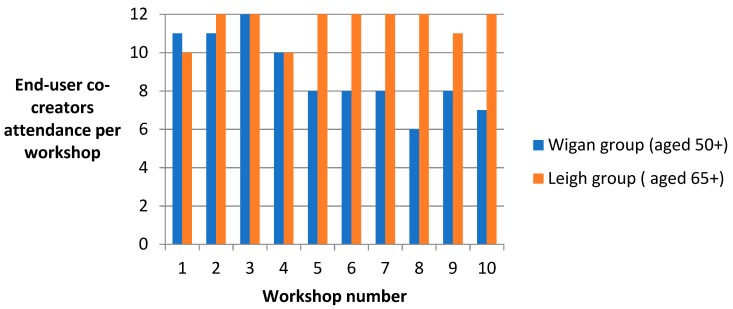
Attendance rates of end-user co-creators.

The attendance of end-user co-creators is reported in Figure 1. Within the Wigan group, the average attendance per session was nine, whilst the Leigh group averaged almost 12. Over the course of the programme, retention rate was 92%, with two participants withdrawing from the Wigan group due to ill health and injury. 

### 3.3. Recommendations for Change and Improvement

The co-creators developed recommendations that were synthesised into four themes: localised strategies for awareness raising, recruitment, accessibility and evaluation. These, along with practical ways to implement them, are reported in Table 3.

#### 3.3.1. Localised Strategies for Awareness Raising Recommendations

##### Promote the UK Physical Activity Guidelines and Locally Available Physical Activity Opportunities

Co-creators identified the need for improved promotion of the physical activity guidelines and targeted services that would enable community residents to be more active. While promotion should target middle aged and older adults, both groups felt that strength and balance, along with wider physical activity guidelines, should be targeted throughout the course of life. Co-creators were unaware of any local promotion of the physical activity guidelines: *“The guidelines were a surprise to me, didn’t know they existed”* (Female, 64 years, Leigh group). They had visited general practice surgeries, pharmacies and leisure centres and saw no evidence of the guidelines being promoted: “*Until last week I didn’t know there were any government guidelines for physical activity—I’m surprised by the emphasis on strength”* (Female, 55 years, Wigan group).

The Wigan group emphasised that the physical activity guidelines should be promoted everywhere and featured in the local council community magazine: *“The guidelines are not known by many—physical activity guidelines should be promoted everywhere”* (Female, 62 years, Wigan group). Further, co-creators suggested promotion of guidelines within a range of different settings (beyond leisure centres) including general practice surgeries, pharmacies, hospital outpatient and physiotherapy departments and anywhere with a waiting area: *“I haven’t seen them (the physical activity guidelines) displayed on the TV screens in the doctor’s waiting area—is this a missed opportunity?”* (Female, 64 years, Leigh group). Other suggestions included supermarkets, community centres, post offices, hairdressers and on buses and trains. 

Regarding the content of promotional material, the Wigan group highlighted the importance of keeping the message simple, tackling peoples’ fear about exercise and using real life case studies that people could relate to: *“We need eye catching posters!”* (Female, 61 years, Wigan group). The Leigh group emphasised the importance of publicising what is already available, as they did not appreciate the variety of activities that were already on offer: *“As a group we were amazed and surprised by the number and range of places and activities for all age groups and abilities”* (Female, 64 years, Leigh group). Further, the need to use a variety of positive images to represent older people as well as infographics to simplify guidelines were thought to be of particular benefit.

##### Encourage Health and Social Care Services to Play an Active Role in Promoting the UK Physical Activity Guidelines

Both groups agreed that general practitioners needed to be more involved in the promotion of the guidelines, identifying that these are the health professionals that most ageing and older adults would interact with: *“It needs to be a practice approach, not just relying on the practice nurse”* (Female, 73 years, Leigh group). However, there was a view that most general practitioners were not aware of the guidelines as they *“never mention physical activity”* (Female, 65 years, Leigh group), and in particular, the benefits it can have on wellbeing: *“GP doesn’t tell us it’s [physical activity] better than tablets”* (Female, 65 years, Leigh group).

The groups identified two ways that professional groups could increase awareness of the guidelines with those who are ageing. Firstly, as part of routine health checks that this group often get (for example with general practitioners or practice nurses), it was suggested that including Functional Fitness Tests [10] to specifically emphasise the importance of strength and balance would be an improvement. Second, the Leigh group suggested awareness training for health professionals around the physical activity guidelines: *“Other staff should take on the role of referring for physical activity*” (Female, 73 years, Leigh group). The aim of this training would be to facilitate a culture shift and support the development of a clear patient pathway and consistent referrals into other initiatives, including volunteer outreach programmes or to existing leisure services.

##### Working Collaboratively to Implement Recommendations over Time

The Wigan group felt that there needed to be a more joined-up approach between their local council, leisure service providers and healthcare services to promote healthy living and actively promote the benefits of the physical activity guidelines: *“I would like a commitment from stakeholders and would like to highlight Wigan Borough’s poor health stats”* (Female, 61 years, Leigh group). Recognising that the needs of end-users should be voiced during the implementation of recommendations, a number of the participants re-iterated their willingness to be involved and to support this: *“The movement also needs to be from us, not just the stakeholders”* (Female, 61 years, Leigh group).

A follow-up meeting with both groups (together) took place six months post-engagement to receive an update on what progress had been made by stakeholders (local council, leisure service providers and healthcare services) and what further plans were to be taken forward. The co-creators were unanimous that they did not want this process to be tokenistic, and instead wanted a commitment to bring their recommendations into practice: *“In 12 months’ time, what will you have in place to make this all worthwhile?”* (Male, 71 years, Leigh group).

#### 3.3.2. Recruitment Recommendations

##### Recruit, Train and Support Volunteer Champions to Provide a Physical Activity Outreach Programme

The Leigh group proposed the development of an outreach programme for volunteer champions who could visit different settings within the community to promote (and potentially lead) physical activity and other health and wellbeing initiatives: *“I have shared my learning—telling others of simple exercises they can do within the home and handing out resources to help this, this had a positive effect”* (Female, 65 years, Leigh group). This could include a programme of practical taster sessions in shopping centres, sheltered housing schemes, residential homes and with other community organisations: *“I would like to share information with other groups in the borough”* (Male, 70 years, Leigh group).

The group were adamant that this programme should be resourced for at least 3–5 years and work alongside existing non-governmental organisations to add value to and maximise the existing services available. Volunteers could initially be recruited through the New Tricks research groups. However, it was acknowledged that additional resources would be required to co-ordinate and support the volunteers, in addition to providing sufficient training for these individuals.

##### Employ Staff and Volunteers as Older ‘Ambassadors’ in Leisure Centres to Promote Age-Friendly Developments

The Wigan group identified a suggestion for the development of an older ‘ambassadors’ programme within the leisure centres. It was agreed that these individuals would have a better understanding of the needs of their peers, as well as being able to act as role models to encourage new participants: *“All promotional pictures are of young, muscly people—no role models”* (Female, 53 years, Wigan group). In addition, they felt that the involvement of older ambassadors could positively influence the approach and practice of other staff working within the facilities, and support other ‘age friendly’ improvements (in particular, the choice of music and volume within the gyms): *“They need to offer times when there is no music or 50+ friendly times (to encourage interaction)”* (Male, 53 years, Wigan group). This idea had emerged through the Wigan group’s experience of using gyms and other classes over the duration of the engagement programme. They proposed seeking out people who have a lifetime of fitness behind them as well as identifying people who have got fitter in later life and can demonstrate the health benefits they have achieved. However, if volunteers rather than paid staff were recruited to be older ambassadors, then incentives should be implemented to acknowledge their contributions.

In contrast, the Leigh group identified the need for training of exercise and fitness instructors to become more age-friendly (in their language and communication) and to be good role models: *“I would like emphasised the importance of having age friendly leisure staff who are supportive and help with using equipment (we don’t have so many at the moment)”* (Male, 63 years, Wigan Group). Both groups felt that the current gym staff could be more attentive to customer needs: *“I was in the gym last week and there was a hue and cry, almost a mild uprising, by quite a few older people about the ’racket’ from the PA system. One gentleman had to take out his hearing aids to cope”* (Male, 63 years, Wigan Group).

#### 3.3.3. Accessibility Recommendations

##### Produce Accessible Physical Activity Information Including the Benefits of Strength and Balance

Both groups identified the need to improve the accessibility of high quality information around the physical activity programmes (for example whether they improved strength, balance and/or endurance) and services available, noting the limited information visible on local websites. The groups recognised the challenges of getting the right level of information and the complexity that might accompany this. The Wigan group emphasised the importance of keeping the information simple, fun and friendly through using cartoons. The Leigh group proposed an approach to provide a “*simple to navigate layered way of accessing information—i.e., just enough information initially so that people aren’t overwhelmed, but an easy way of being able to find out more information”* (Female, 55 years, Wigan group). Both groups felt the use of video clips of activities would be useful in helping people understand what the class was about, as well as helping them to assess the intensity of activity and whether they felt it was appropriate for them. These could be captured and demonstrated by New Tricks group participants—providing more age specific role models. This approach could be achieved effectively online, but should also be available as a hard copy for individuals who do not have access or cannot easily access or navigate the internet and computers. These non-digital versions could be available at local services and printed off at the request of the individual on an ad hoc basis: *“At the moment we are just told to go online to access information*” (Female, 52 years, Wigan group). 

Central to improving the quality of information available, both groups were keen to see the introduction of a grading or rating system (such as a traffic light or star rating system) so that people could understand the strength and balance and other benefits of the different activities available.

##### Explore Different Pricing Options and Incentives for Older Adults’ Physical Activity Participation

Co-creators felt strongly about the need to try and incentivise people to become more active: *“It’s not enough to just tell them about what’s available*” (Male, 63 years, Wigan group). Both groups wanted to see more imaginative approaches to pricing options for leisure use, including incentives through regular participation. The Wigan group felt that older adults miss out: *“They used to have concessions, but not anymore”* (Male, 53 years, Wigan group), and local services could experiment with different pricing options for targeted sessions for older adults: “*There should be heavily discounted or free membership for pensioners to use the gyms at quiet times, when few people are in*” (Male, 53 years, Wigan group). It was also suggested that reduced price sessions could be linked to a loyalty card—*“better discounts the more often you attend*” (Male, 53 years, Wigan group)—or even offering by a discount to members who encourage someone else to sign up. 

Similarly, the Leigh group suggested discounted membership for those on benefits and rewards for people who reached a level of physical activity participation (similar to incentives used by weight-loss services). Co-creators also suggested ‘promotion months’ where people could try out different activities for free.

##### Provide Simple Exercises That People Can Do at Home or in Community Settings

Both groups had been encouraged by simple activities that you could do at home [21], and felt that this was an important idea to share more widely. These activities could help to increase confidence and steadiness, or indeed could be done if people could not attend specific activities because of time commitments or did not want to visit the gym: *“My light bulb moment was [Facilitators name] information—how we can do simple everyday activities to improve or strength and balance”* (Female, 55 years, Wigan group). The Leigh group suggested that the local offer needed to include simple exercises that could be easily built into a daily routine, such as: *“standing on one leg whilst cleaning your teeth”* (Female, 65 years, Leigh group). The group suggested the provision of free resources and practical guidance on how to do the exercises and use any accompanying equipment: *“Simple activity aids, stretch bands and hand squeeze balls for everyone!”* (Male, 53 years, Wigan group). Such activities could be promoted or available in general practice surgeries, sheltered housing and care homes, so that everyone could achieve some sort of strength and balance activity.

##### Improve the Accessibility of Strength and Balance Programmes

The Wigan group identified that most targeted physical activity programmes for older adults or people with chronic health needs only take place in the daytime. They recommended more opportunities be made available in the evenings and at weekends for people with work commitments: *“Frustrated at the lack of evening classes. Most classes during the day—can’t attend due to commitments”* (Female, 60 years, Wigan group). Sessions should also be more widely available in community locations, not just in leisure centres, and should be accessible, offering car parking with close proximity to bus routes: *“Prefer to go local but evening sessions are not available”* (Female, 53 years, Wigan group).

Regarding adaptability, co-creators emphasised that it was important to ensure there were enough adapted activities to be accessible by the majority of older people, including those with conditions that make it difficult for them to engage in some mainstream activities (due to their limitations). The tailoring of activities needs to be considered in the provision of local programmes: *“Several of [local leisure service’s] adapted sessions don’t actually run (e.g., Wheelchair Rugby) or they’re just a time limited period for each individual. [Local leisure service] should make more activities ‘inclusive’ so people with a range of disabilities or conditions can join their friends in an activity and not be side-lined”* (Male, 66 years, Leigh group).

#### 3.3.4. Evaluation Recommendations

##### Monitor and Evaluate Strength and Balance Programmes and Their Impact on Wider Community Awareness

The Leigh group discussed that local leisure service providers should monitor and evaluate physical activity interventions long-term (to ensure that if they say they improve strength, they do). They should also evaluate whether they are engaging more older people over time in these programmes. Further, it was suggested that ongoing consultation could be undertaken with people who both attend and drop out of the programmes to find out what influences their decisions: *“There should be ongoing evaluation of all activity programmes of their effectiveness and who attends them...the big challenge is to engage with people who don’t currently take part in any activities”* (Female, 70 years, Leigh group).

The Wigan group concurred that it would be beneficial to do a survey with local residents to measure if there had been any change in awareness around knowledge of physical activity guidelines and the programmes available.

##### Satisfaction of Engaging in the Co-Creation Process

There was a high level of satisfaction with the engagement programme, reflected in the high level of attendance (85%) and retention (92%) of participants over the duration of the programme. Evaluation feedback demonstrated that: (1) 78% strongly agreed and 18% agreed (96% total) that they had gained new knowledge and skills from the workshops; (2) 81% strongly agreed and 15% agreed (96% total) that they enjoyed participating in the workshops and (3) 77% strongly agreed and 15% agreed (92% total) that they were able to contribute to the workshops. 

Further, the co-creation programme appeared to be very successful in manifesting ownership within both groups. For example, co-creators wrote a ‘jingle’ and a poem to describe the importance of strength and balance and the value of attending the workshops [22], in addition to a shared desire for the recommendations to be taken forward: *“We hope this is not just a 10 week course and that our ideas will be considered and used to shape things in the future”.* Particular elements of the process that co-creators spontaneously reported enjoying included the scientific input: *“The presentation from [name]—the data he showed us was quite thought-provoking. Physical activity becomes more important as we age”* (Male, 66 years, Leigh group); working together as a group: *“We have really enjoyed the sessions and will really miss the social side now it’s finished”* (Male, 70 years, Leigh group) and increased knowledge: *“It’s raised my awareness of the physical activity guidelines—there were so many things that I didn’t know, and I’ve put my learning into practice”* (Male, 70 years, Leigh group).

## 4. Discussion 

The purpose of this study was to co-create with ageing and older adults, recommendations to redesign and promote leisure services with a focus on MBSBA. To the knowledge of the authorship team, this is the first time that a co-creation methodology has been used to inform service redesign specifically targeting strength and balance opportunities to encourage individuals to meet these components of the physical activity guidelines. Co-creation has the potential to enhance the quality of service provision and improve the effectiveness of programmes [12,13], and therefore was utilised here to ensure that local services are tailored to the needs and preferences of that population. Co-creation (to improve local service provision) appears to be appealing to ageing and older adults, given the limited recruitment strategies required to gain a heterogeneous sample of end-users. Overall, engagement and adherence to the co-creation workshops was high (retention rate of 92%), with co-creators providing a range of recommendations and ideas to improve the delivery of local services.

The first key theme that emerged from the workshops was the local promotion of guidelines. Co-creators acknowledged a lack of awareness of the PA guidelines, and, more specifically, most were unaware that strength and balance guidelines existed, often leading to these being referred to as the ‘forgotten guidelines’ [3]. Co-creators suggested that promotional campaigns could be undertaken at a local level across a variety of environments and settings to reach the widest possible range of the local population, such as by utilising promotional material across sheltered housing, supermarkets, public transport and community centres. This would provide the opportunity to reduce health inequalities and improve physical health literacy in often hard-to-reach segments of society [23]. Considering the beneficial effects of physical activity on wellbeing, the co-creators were surprised that healthcare settings (including general practice, pharmacies and physiotherapy clinics) had no promotion of these guidelines. This was unsurprising, considering recent surveys have shown that 16% of physiotherapists [24] and 20% of general practitioners [25] do not know the guidelines. As these settings provide a platform for healthcare professionals to prompt behavioural change within this population, more work is needed to upskill these professionals to take advantage of these opportunities.

A second key theme that emerged was recruitment, with co-creators commenting that voluntary ambassadors from the same demographic could raise awareness and engage individuals to be more active. Co-creators recognised that this peer-support role could provide a support mechanism to people newly attending services, and two voluntarily declared an interest in providing this function. Interestingly, they mentioned this independently of the existing evidence base describing the importance of the peer-support role in uptake and adherence to behavioural change programmes [26]. It is important to note that this was not a topic covered during the scientific input workshop. However, despite being a voluntary role, they emphasised the importance of adequate resourcing to support the sustainability of this role, for example, appropriate coordination and training [27]. An additional function that could be provided by this role is collaboration with exercise instructors regarding their interactions with older adults, to improve attendees’ experiences and, subsequently, potential maintenance of changed behaviour [28].

Considerations around accessibility formed the basis of their third theme. The co-creators provided suggestions that, again, were consistent with previous research, such as reducing financial barriers to physical activity participation [29]. They were particularly mindful of the impact health inequalities could have on engagement, especially when considering the relationship between deprivation and inactivity [30,31]. Ideas that emerged to tackle this issue included a traffic light system, allowing individuals not just to visually recognise which activities had an emphasis on strength and balance, but also to highlight activities which were appropriate for those with particular medical conditions/disabilities. A similar approach to health literacy is used in the food industry, although there is concern about usage and understanding amongst those in lower socio-economic groups [32]. Further ideas, including discounts and free taster sessions, were seen as key facilitators to uptake. Interestingly, they also suggested the use of incentivisation for those who achieve particular activity levels as a mechanism to improve maintenance. This has been used successfully for other health behaviours in deprived groups (such as smoking [33] and school-based parenting programmes [34]), and may be valuable to adopt here.

One of the main findings here that was congruent with the literature was the element of choice. For example, co-creators commented that access to information regarding available activity opportunities should be available in both digital and non-digital formats. This was one strategy to ensure equitable accessibility of information, particularly for those who are not digitally connected [13,35]. However, an element of choice that contrasted with published literature was the timing of exercise sessions for older people. While previous work has suggested that mid-morning and early afternoon sessions are more likely to attract older people [36], the co-creators highlighted that choice of timing is important, particularly considering the increasing number of older adults in employment during the daytime [37]. This warrants a reconsideration of service provision regarding times of activities during the week, evenings and weekends. Further, it highlights one particular strength of co-creation towards developing localised solutions for local needs and preferences. 

A key consideration for co-creation processes is ensuring that the developed outcome(s) is mutually beneficial to all stakeholders [16]. Here, the co-creators appeared to be highly satisfied with their engagement; there was an average of 85% attendance across sessions and strong agreement that they had developed skills and knowledge over the workshops (96% strongly agreed). Particular areas identified as being enjoyable included the social elements and group cohesion, the scientific input from researchers (such as the FFMOT) and self-reported improvements in wellbeing. Examples of ownership manifested within the co-creation group (92% strongly agreed they were able to contribute in the workshops), for example, one co-creator wrote a poem to describe the purpose of the workshops, whilst others volunteered to become ambassadors for the leisure trust. However, they also recognised that they did not want their recommendations to be ignored. Instead, there was an expectation that they would be implemented and disseminated in the future. As such, there is a responsibility for the instigators of co-creation processes to ensure that appropriate consideration and resources are allocated so recommendations can be implemented [16]. 

There were several strengths to this study. First, the satisfaction and ownership of co-creators was emphasised by the high retention and adherence rates. Second, the co-creation facilitators received training from experts in co-creation, therefore ensuring that appropriate governance was applied to the process, such as sampling considerations, equality of contribution and process validity [16]. However, there were some limitations that should be acknowledged. First of all, individuals who volunteer for such processes may not necessarily be representative of the wider population; however, the research team purposefully sampled those who applied to cover a range of characteristics, including age, gender, ethnicity and medical conditions. Furthermore, as workshops were not audio recorded and transcribed, it is possible that not all recommendations were fully captured here. However, member checking occurred at the end of each session to ensure that all captured ideas were representative of the thoughts of the group, and the final report was shared with the co-creators to ensure that all recommendations were included.

## 5. Conclusions

This study demonstrated the feasibility and benefits of engaging local ageing and older adults to co-create recommendations for raising awareness about physical activity guidelines for health, and for better leisure service provision to facilitate their meeting these recommendations. In this context, considerations specific to strength and balance provision included: (1) localised strategies for awareness raising, (2) recruitment of volunteer champions to increase uptake and maintenance, (3) accessibility of activities, including what they are and when they are, and (4) mindfulness of adaptations necessary to reduce health inequalities. Future research should aim to implement these recommendations to evaluate what changes, if any, they make to awareness, uptake, adherence and sustainability of service provision.

## Figures and Tables

**Table 1:** Overview of co-creation workshop content.

Workshop	Key Discussion Areas	Additional Tasks
Week 1: Induction/overview	Introductions from facilitators and participants	Programme paperwork and questionnaires
Week 2: Research and evidence	Physical activity guidelines	Expectations of the programme
Week 3: Research and evidence	Strength and balance and ageing	Functional Fitness MOT tests [10]
Week 4: Research and evidence	Barriers and enablers for being more active	Practical ideas for incorporating strength and balance
Week 5: Research and evidence	Assessing the value of activity choices for strength, balance or other outcomes	Supplemented by an overview of the leisure offer (with emphasis on strength and balance activities)
Week 6: Practical experience of programmes on offer	Opportunity to try out activities and find out more	
Week 7: Identifying areas for improvement	Exploring ideas and recommendations for local service provision	Exploring ideas and recommendations for promoting services and guidelines
Week 8: Exploring how groups can influence change	Exploring opportunities to influence positive change through the programme	Planning for stakeholder session and prioritising key recommendations/learning
Week 9: Presentation to external stakeholders	Sharing recommendations and ‘light bulb’ moments	Personal journeys and contributions
Week 10: Reflections on programme and next steps	Feedback on stakeholder event and final matters to raise	Feedback on programme overall and plans moving forward (personal/as a group)

**Table 2:** Profile of end-user co-creators (*N* = 24).

Characteristics	Number (*N*)
Gender, *N* (%)	
Female	14 (58)
Male	10 (42)
Ethnicity, *N* (%)	
White British	22 (92)
Black British	1 (4)
Asian British	1 (4)
Living with chronic health condition, *N* (%)	14 (58)
Living with a disability	12 (50)
Physical activity levels, *N* (%)	
Inactive (less than 30 min of physical activity per week)	5 (21)
Fairly active (30–149 min of physical activity per week)	14 (58)
Active (150 min of physical activity per week or more)	5 (21)
Socio-economic status *N* (%)	
Quintile 1 (Least affluent)	0 (0)
Quintile 2	2 (8)
Quintile 3	7 (29)
Quintile 4	6 (25)
Quintile 5 (Most affluent)	7 (29)
Not reported	2 (8)

**Table 3:** Themes and recommendations for change.

Theme	Recommendations
Localised strategies for awareness raising	Promote the UK Physical Activity Guidelines and locally available physical activity opportunities Encourage health and social care services to play an active role in promoting the UK Physical Activity Guidelines Working collaboratively to implement recommendations over time
Recruitment	Recruit, train and support volunteer champions to provide a physical activity outreach programme Employ staff and volunteers as older ‘Ambassadors’ in leisure centres to promote age-friendly developments
Accessibility	Produce accessible physical activity information, including the benefits of strength and balance Explore different pricing options and incentives for older adults’ physical activity participation Provide simple exercises that people can do at home or in community settings
Evaluation	Improve the accessibility of strength and balance programmes Monitor and evaluate strength and balance programmes and their impact on wider community awareness
